# Assessment of mental health among nursing staff at different levels

**DOI:** 10.1097/MD.0000000000019049

**Published:** 2020-02-07

**Authors:** Man-Li Du, Wan-Xin Deng, Wen Sun, Ching-Wen Chien, Tao-Hsin Tung, Xin-Chun Zou

**Affiliations:** a Huadu District of Guangzhou Maternal and Child Health Hospital (Huzhong Hospital), Guangzhou, China; b Institute for Hospital Management, Tsing Hua University, Shenzhen Campus, China; c Department of Medical Research and Education, Cheng-Hsin General Hospital, Taipei, Taiwan; d The Affiliated Stomatology Hospital of Kunming Medical University, Kunming City, China.

**Keywords:** depression, hostility, mental symptoms, nursing staff

## Abstract

To assess the mental health of nurses and to find the post responsibility and psychological status of clinical nurses.

A total of 447 nursing staff at different levels in a teaching hospital was assessed by nursing post responsibility scale and mental symptom checklist (SCL-90) then compared with each other. The study period was from April 1, 2018 to April 30, 2018.

There was a positive correlation between the responsibility of post and interpersonal relationship (r = 0.11, *P* < .05), depression (r = 0.10, *P* < .05) and hostility (r = 0.10, *P* < .05). Post risk was negatively correlated with somatization (r = −0.10, *P* < .05), job involvement scope and communication ability were negatively correlated (r = −0.11, *P* < .05). Based on the multiple linear regression, knowledge and skills (β = −0.20, *P* = .02) and risks of the post (β=0.20, *P* < .01) both significantly related to SCL-90 total score.

In conclusion, knowledge and skills and risks of the post associated with mental health of clinical nurses. The sustainable development of nursing post responsibility requires healthy physiological and mental health.

## 1 Introduction

The mental health of nursing staff is one of the valued topics of nursing research. In recent years, great changes in society have been made. Higher demands on the skills and quality of nursing staff.^[1]^ Nursing staff are strictly required to conduct level nursing post duty training to improve their professional skills. Post responsibility is based on the results of post analysis, according to the necessary criteria, the nature of the work, intensity, responsibility, complexity and the required qualifications and other status differences in a comprehensive assessment.^[2]^

Nursing staff is the key group to provide and protect health for people. Their mental health is closely related to quality nursing service. How to coordinate the mental symptoms of nursing staff and mobilize nursing staff is an important task for hospital leaders to solve the problems of nursing staff, to fully explore their potential, to improve their comprehensive quality and to provide patients with diversified, multi-level and individualized health services.^[3]^ In nursing management, the positive psychological attitude and good atmosphere of innovation of nursing staff are helpful to promote nurses’ divergent thinking, to improve their sense of work belonging, and to improve the quality of nursing service.^[4]^ Therefore, this hospital-based study is conducted to explore the assessment of mental health among nursing staff at different levels.

## 2 Methods

### 2.1 Setting and participants

The purposive sampling for this hospital-based study was conducted in Guangdong, China from April 1, 2018 to April 30, 2018. A total of 447 nursing staff were selected at a fully certified regional and teaching hospital. Based on the nursing career development and the actual situation in the study hospital, the nursing functional promotion post consisted of four levels, that is, N1 (primary nurse): 1–3 years; N2 (senior nurse): 3–5 years; N3 (nursing leader): 5–8 years; N4 (specialist nurse): 8 years or more. In China, from the medium and long term Plan for the Construction of Professional and Technical personnel (2010–2020),^[5,6]^ the proportion of professional and technical personnel in accordance with the high, middle and primary level is 10:40:50.

In this study, all participants completed a questionnaire pack at one time point. In addition, for the calculation of the sufficient statistical power, a sample size of 447 achieves 95% power using effect size 0.05 and 7 predictor with a significance level (alpha) of 0.05.^[7]^

### 2.2 Procedures and ethical considerations

This study was approved by hospital's institutional review board (gzhdfy2018hl01). All procedures were performed in accordance with the guidelines of our institutional ethics committee and adhered to the tenets of the *Declaration of Helsinki*. All subjects information was anonymous.

First, researchers contacted and explained the research procedures and emphasized that participants’ responses were anonymous and confidential. Then the selected subjects were informed by the researcher that the survey was voluntary, the results would remain anonymous, and there were no right or wrong answers. It was also emphasized that the investigation was being done only for the purpose of studying participants’ subjective perceptions. Finally, study subjects were asked to sign a letter of authorization before completing the self-report questionnaire.

### 2.3 Measurement instruments

#### 2.3.1 Nursing post responsibility scale

Job responsibilities, including knowledge and skills, post responsibilities, post risks, job complexity, scope of work and communication skills and innovation skills are 6 dimensions. The scale has 36 evaluation factors; each item has the corresponding post representative.^[8]^

#### 2.3.2 Mental health assessment tool

The Symptom Check List-90 (SCL-90) is a self-report clinical rating scale. From the clinical and academic viewpoint, the SCL-90 not only has been designed as a general measure of psychiatric outpatient symptomatology, but also was developed with primary emphasis on validity as a criterion measure in clinical trials where focus is on the relative efficacy of psychotherapeutic agents.^[9]^ There are 9 factors in the SCL-90, including somatization, obsessive symptoms, interpersonal relation, depressed, anxious, hostility, terrifying, bigoted, and mental degeneration.^[9]^ Each item on this scale was measured on a 5-point Likert scale, with 1–5 indicating the severity of the symptoms as follows: “1: no”, “2: light”, “3:moderate”, “4:quite heavy”, and “5:severe”. A higher answered score reflected a higher frequency and intensity of symptoms.^[10]^ In this study, the total score greater than 160 was considered to be positive for psychological symptoms.^[11,12]^

### 2.4 Data analysis

Statistical analyses were performed using SPSS 20.0. Subjects’ characteristics were reported as means and standard deviation (SD) for continuous variables and proportions for categorical variables. In the univariate analysis, the independent t-test method or χ^2^-test was adopted and the Pearson's correlation coefficient was used to assess the association of the mean value. The multiple linear regression model was used to assess the independent effects of relevant factors on SCL -90 values after controlling for the covariates. A two-sided *P* value < .05 was considered statistically significant.

## 3 Results

A total of 447 clinical nurses (100% response rate) were assessed with self-rating scale for nursing posts and psychological symptoms. Table 1 shows that number of N1, N2, N3, and N4 were 213 (47.7%), 144 (32.2%), 51 (11.4%), and 39 (8.7%), respectively. The mean ± SD total points of nursing duty score and SCL-90 score were 458.72 ± 95.16 and 124.45 ± 34.43.

**Table 1:**
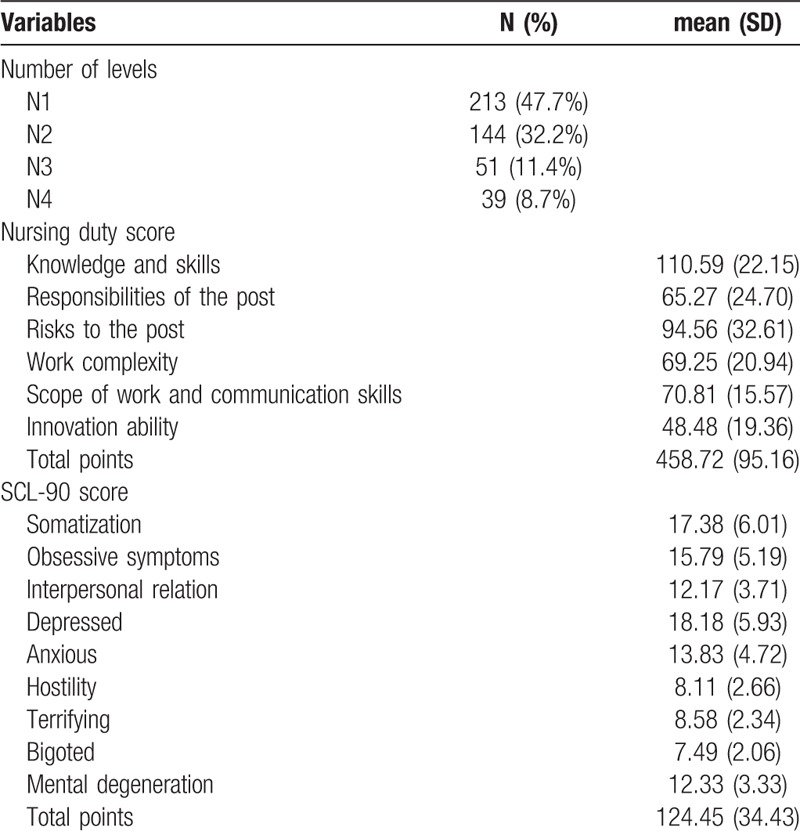
Baseline information of nursing post responsibility and SCL-90 (n = 447).

Table 2 shows that the nursing post responsibility is correlated with the interpersonal relationship, depression and hostility in SCL-90 (*P* < .05), and there is a positive correlation between the responsibility of the post and the interpersonal relationship, depression and hostility in SCL-90 (*P* < .05). There was a negative correlation between post risk and somatization symptom (*P* < .05), more significant correlation with total score of SCL-90 (*P* < .05), and negative correlation between job involvement scope and communication ability and anxiety and hostility (*P* < .05).

**Table 2:**
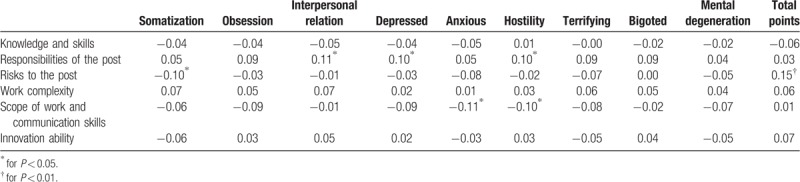
The Pearson's correlation coefficient between nursing post responsibilities and SCL-90 scores.

From Table 3, the proportion of N4 in the positive signs of SCL-90 is the highest, and the proportion of N4 in the positive signs of SCL-90 is increasing with the higher level. In addition, as mention in the method, the SCL-90 total score greater than 160 was considered to be positive for psychological symptoms. Table 3 also indicates that the subjects with SCL-90 total score ≥160 had higher scores of risks to the post than those <160 (108.49 ± 35.38 vs 92.69 ± 31.81, *P* = .001).

**Table 3:**
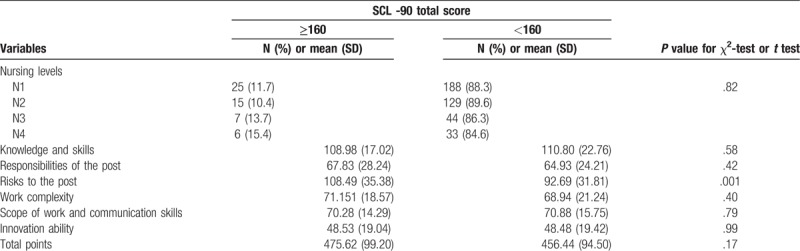
Positive symptoms of SCL-90 in all levels of nursing staff and job responsibilities (n = 447).

The effects of independent factors of SCL-90 total score were examined by the multiple linear regression model. Table 4 shows that knowledge and skills (β = −0.20, *P* = .02) and risks of the post (β = 0.20, *P* < .01) both significantly related to SCL-90 total score after adjustment for confounders.

**Table 4:**
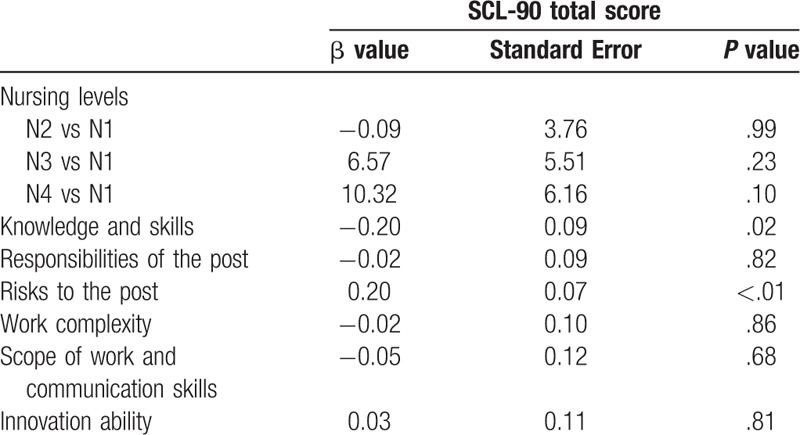
Multiple linear regression analysis of the total score of post responsibility and level nursing staff in SCL-90 (n = 447).

## 4 Discussion

### 4.1 Clinical implications

In China, the SCL-90 is one of the important assessment tools for mental health and used widely in clinical mental health practice.^[10]^ This tool uses have ranged from evaluation of the mental health in various groups, as well as comparisons of different symptom groupings.^[13]^ This study showed that 11.9% of the nurses’ score of SCL-90 is estimated more than 160, indicating that they possibly had unhealthy mental tendencies, and medical staff had problems in interpersonal relationships, depression, hostility and anxiety. It also implied that if the clinical nurses with symptoms of depression and hostility, in addition to understand and fully affirm their work achievements, they may conduct individual mental health counseling. If the mental symptoms do not relieve and gradually affect daily life, clinical nurse staff may further reach to professional psychological counseling and treatment suggestions.

The physical, cognitive, and emotional demands inherent in nursing practice have been indicated to have a negative effect on the physical and psychological well-being.^[14]^ Nursing staff's mental health is worse than the general population, especially in ages less than 40-year nurse group have higher incidences of mental health problems.^[15,16]^ Previous academic studies used SCL-90 accessing nursing mental health in various populations also found that the Chinese nurse group faces greater occupational pressure and is different from the psychological status of ordinary adults.^[17–20]^ There is consistent in our study when compared with other researches. In addition, the symptoms of depression and hostility were obvious among nurses at different levels. The higher the nursing posts, the more medical comprehensive quality education they accepted, the higher their professional skills, the higher their social status, the higher their professional competitiveness, the higher their professional development career, the higher their professional skills, and the higher their scientific research and teaching work. High workload, high repeatability, the risk of disease infection and the improvement of medical level require higher professional competence of medical staff, so that nurses feel greater occupational stress.^[21,22]^ Physical and mental injury, increased turnover rate, affect the stability of the medical team, increase medical risk. High intensity of work can cause bad mental state, increase depression and hostile mood, meanwhile induce mental illness in long turn, so it is very meaningful to enhance the mental health of nurses.

From the viewpoint of clinical practice, mental health is an issue that needs preventive interventions due to the stressors that subjects are exposed to in the workplace could inflict mental pain and increase the risk of incident physical health problems.^[23]^ Mental health refers to the best state of mind and body of each individual under subjective and objective conditions. Mental health does not mean absolute perfection.^[24]^ The nature and characteristics of nursing work determine that nurses’ mental symptoms are becoming more and more prominent and complex, and that nurses face stress or threat in clinical practice in their duties. This is a phenomenon that all nursing staff believed is widespread.

### 4.2 Methodological considerations

Several methodological limitations should be considered when interpreting the results of this study. First, the study population is based on a voluntary selection, that is, the participants done not only would potentially introduce self-selection bias, but also Hawthorne effect is inevitable due to the subjects were made a conscious decision to be in the selected study hospital. Voluntary bias could be viewed as that comes from the situation that a special sample could contain only those subjects who are totally willing to participate in the investigation and who participate and know the topic especially interesting are more likely to volunteer for that study, same to those who would like to be evaluated on a positive level.^[25,26]^ Second, we conducted measurements only at a single time point, which might not reflect long-term exposure to the factors related to nurse’ mental health. Third, due to the use of the SCL-90 remains controversial in the study of mental health assessments, further study is needed to determine its reliability and validity in evaluations of Chinese clinical nurses’ psychological health.

Finally, this study only obtained subjects from one teaching hospital in Southern China as the target population. Therefore, the results of this study should not be extrapolated to hospitals in other regions of China. Future studies using random sampling approach of hospitals over wider regions would make the findings more discursive.

## 5 Conclusion

In conclusion, knowledge and skills and risks of the post associated with mental health of clinical nurses. The sustainable development of nursing post responsibility requires healthy physiological and mental health. It is also propitious to establish reasonable planning of human resources and establish self-expectation and self-encouragement standard of nursing staff.

## Acknowledgments

The authors thank the Sunflower Statistical Consulting Company, Kaohsiung, Taiwan for statistical advice.

## Author contributions

**Conceptualization:** Man-Li Du, Ching-Wen Chien.

**Data curation:** Wan-Xin Deng, Wen Sun, Tao-Hsin Tung.

**Formal analysis:** Wen Sun.

**Methodology:** Man-Li Du.

**Resources:** Wan-Xin Deng.

**Writing – original draft:** Man-Li Du.

**Writing – review & editing:** Ching-Wen Chien, Tao-Hsin Tung, Xin-Chun Zou.
